# The effects of spatial and temporal heterogeneity on the population dynamics of four animal species in a Danish landscape

**DOI:** 10.1186/1472-6785-9-18

**Published:** 2009-06-23

**Authors:** Richard M Sibly, Jacob Nabe-Nielsen, Mads C Forchhammer, Valery E Forbes, Christopher J Topping

**Affiliations:** 1 School of Biological Sciences, University of Reading, Whiteknights, Reading, RG6 6PS, UK; 2 Section for Climate Effects and System Modelling, National Environmental Research Institute, University of Aarhus, Frederiksborgvej 399, PO Box 358, DK-4000 Roskilde, Denmark; 3 Centre for Integrated Population Ecology, Department of Environmental, Social and Spatial Change, Roskilde University, DK-4000 Roskilde, Denmark; 4 Department of Wildlife Ecology & Biodiversity, National Environmental Research Institute, University of Aarhus, Grenåvej 14, DK-8410 Rønde, Denmark

## Abstract

**Background:**

Variation in carrying capacity and population return rates is generally ignored in traditional studies of population dynamics. Variation is hard to study in the field because of difficulties controlling the environment in order to obtain statistical replicates, and because of the scale and expense of experimenting on populations. There may also be ethical issues. To circumvent these problems we used detailed simulations of the simultaneous behaviours of interacting animals in an accurate facsimile of a real Danish landscape. The models incorporate as much as possible of the behaviour and ecology of skylarks *Alauda arvensis*, voles *Microtus agrestis*, a ground beetle *Bembidion lampros *and a linyphiid spider *Erigone atra*. This allows us to quantify and evaluate the importance of spatial and temporal heterogeneity on the population dynamics of the four species.

**Results:**

Both spatial and temporal heterogeneity affected the relationship between population growth rate and population density in all four species. Spatial heterogeneity accounted for 23–30% of the variance in population growth rate after accounting for the effects of density, reflecting big differences in local carrying capacity associated with the landscape features important to individual species. Temporal heterogeneity accounted for 3–13% of the variance in vole, skylark and spider, but 43% in beetles. The associated temporal variation in carrying capacity would be problematic in traditional analyses of density dependence. Return rates were less than one in all species and essentially invariant in skylarks, spiders and beetles. Return rates varied over the landscape in voles, being slower where there were larger fluctuations in local population sizes.

**Conclusion:**

Our analyses estimated the traditional parameters of carrying capacities and return rates, but these are now seen as varying continuously over the landscape depending on habitat quality and the mechanisms of density dependence. The importance of our results lies in our demonstration that the effects of spatial and temporal heterogeneity must be accounted for if we are to have accurate predictive models for use in management and conservation. This is an area which until now has lacked an adequate theoretical framework and methodology.

## Background

Population ecology takes the population as the unit of study, identifies factors responsible for population growth or decline, and quantifies their effects. Variations in the circumstances of individuals in time and space (heterogeneity) are generally ignored. However real landscapes rarely approximate to homogeneity, and spatial and temporal heterogeneity are the norm in the fragmented landscapes of the natural world. Thus it is important to know whether and how spatial and temporal heterogeneity affects population dynamics.

Population dynamics often begins by analysing the relationship between a population's density and its growth rate [1,2]. Population growth rate, *pgr *hereafter, is defined as the *per capita *growth rate of the population. The relationship between *pgr *and the natural logarithm of density determines whether a population will return to equilibrium after a disturbance, and the slope of the relationship determines how fast any such return will be. The negative of the slope is referred to as return rate [3] or as the strength of density dependence (e.g. [4]), and is sometimes estimated from the first coefficient in an autoregression analysis (e.g. [5,6]. In discrete generation models a return rate of one per unit time means that a population returns to equilibrium after perturbation in a single time unit in the absence of further perturbations [3]. Positive return rates less than two indicate population stability, and return rates less than one indicate that population density approaches equilibrium smoothly without oscillating (see [6] for further discussion).

Return rates and carrying capacities are key measures in the analysis of population dynamics. Until recently most studies of population dynamics have assumed that both are constant in space and time, and spatial and temporal heterogeneity has generally been ignored. However heterogeneity can affect vital rates (*e.g*., [7-9]) and density dependent processes (*e.g*., [10,11]), for example return rates have been shown to vary with predator density in a tropical damselfish (*Dascyllus fiavicaudus*) [12]. Since heterogeneity is widespread in real landscapes, it is important to know whether and how spatial and temporal heterogeneity affect local carrying capacities and return rates.

Several approaches to incorporating heterogeneity into population dynamic analyses have been taken. The simplest ignores landscape structure and studies resemblances between the autocorrelation coefficients of spatially separated populations. Using this method return rates have been shown to decrease with latitude in grouse populations in North America [13], in caribou and reindeer in Greenland, Finland and Russia [14], and in voles (*Microtus arvalis*) in Fennoscandia and eastern Europe [5,15] but cf. [16]. Return rates of large herbivore populations are increased by temporal heterogeneity in weather, but decreased by spatial heterogeneity in resources in the Rocky Mountains, USA [17]. However return rates of red kangaroos (*Macropus rufus*) did not vary among pastoral zones in South Australia [18]. At the other end of the spectrum landscape ecology provides more realistic treatments of the effects of heterogeneous landscapes on the animals that live there, but has so far little considered their population dynamics. However some progress has been made identifying landscape features that predict species presence, persistence and dispersal [19]; using analytic spatially explicit models to determine population spread rates [20] and growth rates [21]; and using our system to study how landscape framentation affects predator-vole dynamics [22].

There is therefore a need for population dynamics theory that effectively incorporates realistic effects of spatial and temporal heterogeneity. Here we use agent-based models (ABMs) to explore the mechanics and dynamics of four ecologically-contrasting species in a heterogeneous Danish landscape. Spatial variation in local carrying capacity is expected because habitats vary across landscapes (e.g., [23]), but return rates are expected to be invariant unless the mechanisms of density dependence vary. These predictions are largely supported.

## Methods

In this paper population density is described by log_e_(*N*_*t*_), where *N*_*t *_is the number of adult females in a specified area in year *t*; *pgr *is estimated as log_e_(*N*_*t*+1_/*N*_*t*_); return rate as the negative of the slope of the relationship between density and *pgr*; i.e. as – [d*pgr*/dlog_e_*N*_*t*_]_*K *_≡ - [*N*_*t *_d*pgr*/d*N*_*t*_]_*K*_, where local carrying capacity, *K*, is defined as population size in a specified area when *pgr *= 0. In practice the specified areas are 500 × 500 m grid squares as described below.

**Figure 1:**
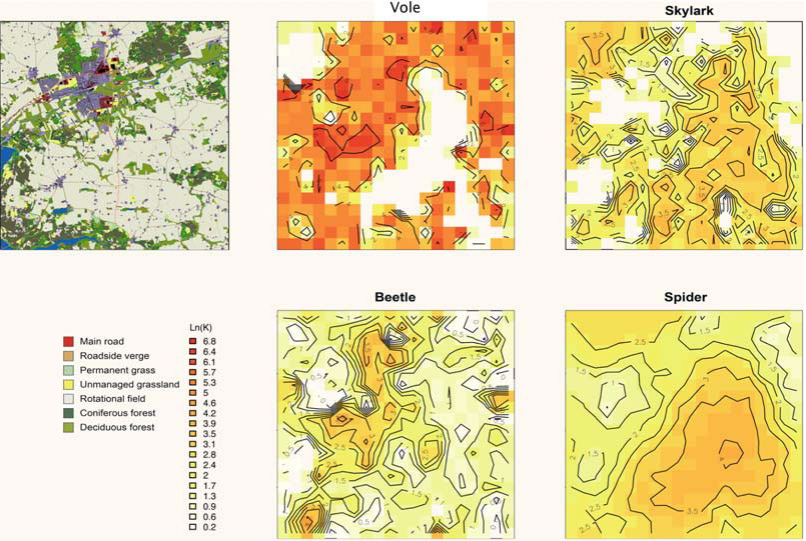
**Maps of the study landscape**. Left hand panel shows the physical map. Remaining maps showing carrying capacities of each species in 1995. Densities (log_e_(K) are indicated by colour (see key). Contours linking similar densities were fitted using R [31].

The study species were *Alauda arvensis *(skylark), *Microtus agrestis *(field vole), *Bembidion lampros *(ground beetle) and *Erigone atra *(linyphiid spider). These were selected because they represent different functional groups, and each has qualities that make them representative of many other species. They are also each of particular interest in the management and conservation of the study landscape. The behaviour and ecology of each species were modelled using agent-based models (ABMs) in a realistic environment. ABMs simultaneously model the independent autonomous behaviours of many interacting agents, here animals. They are used where the factors influencing the behaviour of individual agents are known, but the needed analyses are done at the population level [24]. Thus ABMs provide a means of linking analyses at the individual level to analyses at the level of the population. The ABMs of the study species are described in [25-27]. While the models are necessarily detailed and complex they do incorporate as much as possible of what is known of the behavioural ecology of each species. They simulate the behaviours of numerous autonomous individuals in a 10 × 10-km Danish landscape mapped to a precision of a metre (Figure. 1) using a time step of one day. The behaviours of individuals vary continuously being determined by local geographical features (roads, hedges, habitat type, which in turn depend on plant growth and the actions of individual farm managers and other dynamic features of the Danish environment [28]) as well as weather conditions and interactions with conspecifics. The overall population dynamics emerge from the consequences of interactions among individuals in the model. The four ABMs used have previously been shown to reproduce a wide range of patterns observed in actual field data, both quantitatively (e.g. abundance or sex-ratio against time) and qualitatively where detailed data were not available but qualitative patterns were known. Detailed documentation of the vole and beetle models describing their components down to the level of the source code is provided as a large number of interlinked html documents at . This documentation includes a complete description of the field vole and beetle models together with documentation of central ALMaSS constructs including a class for handling population level functionality and farm and crop management. Documentation follows a combination of the overview and design sections of the ODD protocol [29] with detailed program code comments extracted and integrated using Doxygen [30] and covers the main features of the approximately 70,000 lines of C++ code.

These simulation tools gave us an opportunity to carry out experiments on dispersing animals with complex life histories within Danish landscapes. Simulations were initiated with individuals distributed in the landscape at random, their number being close to the overall carrying capacity of the landscape, and run for 200 years repeating a real 10 year weather data sequence 20 times. This was to allow for the analysis of temporal variation using 'weather years' as explanatory variables in the GLM models described below.

### A GLM population model

**Figure 2:**
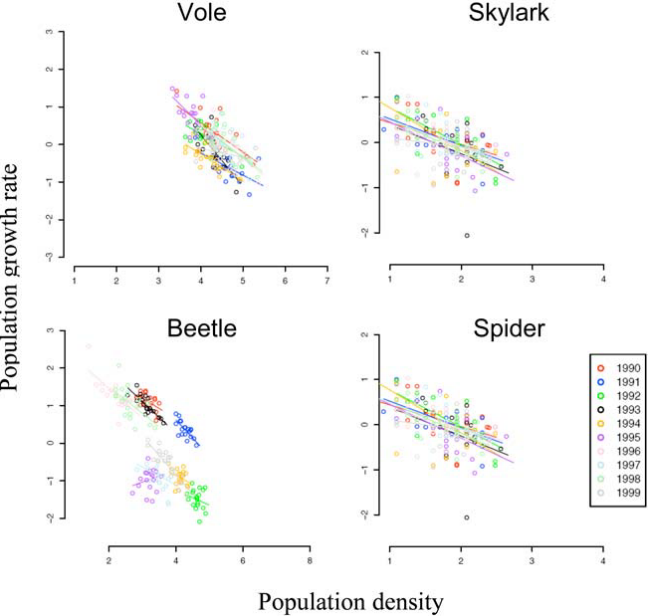
**The effects of weather year on the relationship between *pgr*, y^-1^, and local population density**. Each panel shows a plot of *pgr *vs. log_e_(*N*) for one of the four study species for a randomly chosen grid square. Results for each weather year are indicated by a single colour (see key). Each point specifies the density and *pgr *of one replicate of one weather year.

**Figure 3:**
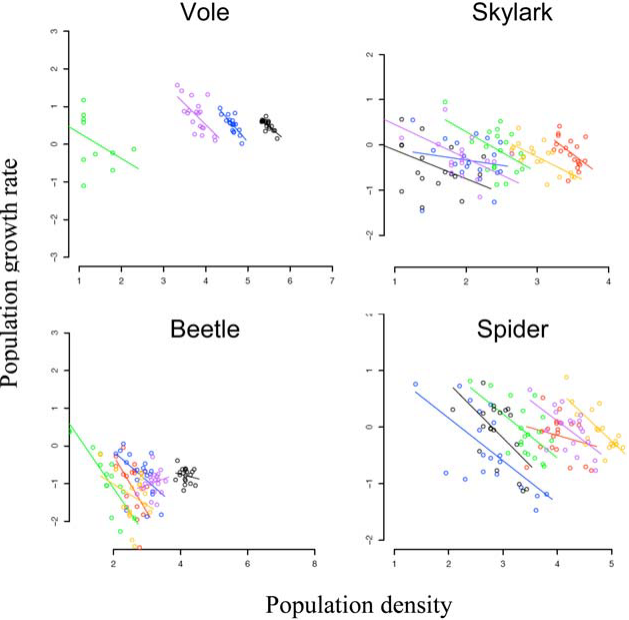
**Spatial variation in the relationship between *pgr*, y^-1^, and population density**. Plots of *pgr *vs. log_e_(*N*) as in Figure. 2 for a randomly chosen weather year, 1995. Colours distinguish each of six randomly chosen grid squares. Mauve indicates the grid square analysed in Figure. 2.

For analysis of spatial variation the landscape was notionally divided into 500 m × 500 m squares in each of which the local population was counted on 1 June each year, and *pgr *calculated. Only 50 evenly distributed non-contiguous squares were entered into the analysis to avoid the possibility of statistical dependence between population sizes in neighbouring squares; however the results were essentially unchanged if all squares were entered. Extensive inspection of how within-square density affected *pgr *showed that the effects of density were linear (examples in Figures. 2 and 3), so for further analysis we used general linear models (GLMs), with the *pgr *for the square as the dependent variable. Population density for each square, defined at the start of Methods, was entered as a covariate, and weather year and square identities were entered as factors. Our design allowed us to analyse the effects on the fundamental *pgr*-density relationship of variation between squares and years. The full design comprised 50 squares and 10 weather years each replicated 20 times, a total of 10000 data per species. However *pgr *could not be calculated for the last year, and data from the first 11 simulation years were excluded from the analyses (burn-in period) since these sometimes yielded populations far from equilibrium, violating the assumption of linear effects. This left a dataset of 50 × (200 - 12) = 9400 for each species. Some datasets were further reduced because empty squares were removed from the analysis. Carrying capacities were estimated as the intersections with the horizontal axes (*pgr *= 0) of the fitted linear *pgr*-log_e_(*N*) relationships for each grid square and year. Analyses were carried out in R [31].

## Results

The effects of weather year and spatial variation on the fundamental relationships between *pgr *and local population density are illustrated in Figures. 2 and 3. The panels within each figure show plots of *pgr *vs. log_e_(*N*) for each of the four species. Figure. 2 shows the effects of variation between weather years, denoted by colours, for a randomly chosen grid square. Note that in all cases density had a linear effect on *pgr *for each weather year, and the slopes of the lines were similar within species. There was not much difference between weather years in voles, skylarks and spiders (points overlie) but weather years were clearly segregated in the beetle. We quantified these effects using GLMs: the resulting summary ANOVA tables are shown in Table 1. The proportion of variance explained by each effect is shown as R^2^. The difference between weather years accounted for only 0.03 – 0.13 of the total variance in the voles, skylarks and spiders, but 0.43 in the beetles (Table 1). This large value of R^2 ^for weather years for the beetles reflects the segregation of lines in the beetle data in Figure. 2.

**Table 1:** Summary ANOVA tables for each species for GLMs regressing *pgr *against population density (log scale), weather years and squares and their interactions.

	Vole			Skylark			Beetle			Spider		
	df	F	R^2^	df	F	R^2^	df	F	R^2^	df	F	R^2^

Density	1	602	0.04	1	630	0.05	1	12810	0.19	1	3036	0.15

Year	9	222	0.13	9	42	0.03	9	3283	0.43	9	276	0.12

Square	45	100	0.29	42	93	0.30	49	320	0.23	49	117	0.28

density*year	9	2	0.00	9	2	0.00	9	34	0.00	9	15	0.01

density*square	43	25	0.07	42	2	0.00	49	4	0.00	49	1	0.00

year*square	378	2	0.04	378	1	0.04	440	4	0.02	441	2	0.03

density*year*square	363	1	0.03	378	1	0.03	434	1	0.01	440	1	0.02

Residuals	6253			7118			7843			7774		

Population density was entered as a covariate, weather years and squares as factors. Empty squares were not included in the analysis and this reduced df in some cases. R^2 ^is the proportion of variance accounted for by predictors and interaction terms.

Figure. 3 shows the effects of variation between grid squares, denoted by colours, for a randomly chosen weather year. Density had a linear effect on *pgr *for each grid square, with similar slopes within species except the vole, where it appears squares showing larger population fluctuations had shallower slopes (this is discussed further below). The lines intersect *pgr *= 0 at carrying capacity, and these equilibrium densities varied widely between squares in each species, as expected since habitats varied across the landscape (Figure. 1, left-hand panel). The proportion of variance in *pgr *explained by grid square was similar for all four species (0.23 – 0.30, Table 1).

The slopes of the lines did not vary with square or year in skylark, beetle and spider, as can be seen from the two-way interaction terms involving density in Table 1. These interaction terms explained ≤ 0.01 of the variance in all species, except that the density*square interaction accounted for 0.07 of the variance in the vole. There were no significant three-way interactions, as can be seen from the F values, which were all one. The implication is that the *pgr*-density relationships of different square and years are essentially parallel in skylark, beetle and spider. Average slopes of all four species are shown in Table 2 and all were less than one (*t*-tests, *p *< 0.001).

**Table 2:** Return rates, y^-1^, for each species obtained as minus the regression coefficient for density.

Vole	Skylark	Beetle	Spider
0.60 (0.01)	0.70 (0.01)	0.65 (0.01)	0.87 (0.01)

Spatial and temporal heterogeneity is accounted for using a GLM performed as in Table 1 but without interaction terms except for year*square. Standard errors are in brackets.

Local carrying capacities of the grid squares are shown in maps in Figure. 1.

## Discussion

Population characteristics are emergent properties of collections of individuals in ABMs as in the real world [24]. It follows that even if individuals are completely deterministic, it is not necessarily true that population characteristics are directly related to environmental characteristics such as weather year or habitat quality in simple one-to-one relationships. In particular, none of the population characteristics revealed in the present analyses were known in advance. Instead they emerged from characteristics of landscapes, weather and individual behaviour reported in field studies and programmed into the ABMs. Thus the revealed population characteristics require explanations. Ideally these will be in terms of the properties of individuals in simple mechanistic relationships, but there is no guarantee such relationships exist. Below, we suggest mechanisms in terms of known and programmed behavioural ecology, and we back up our suggestions where possible by simulation experiments [32] and further analysis of simulation data. However proof of the validity of our conjectures requires in some cases additional experimentation beyond the scope of the present paper.

There was considerable spatial variation in local carrying capacity (Figure. 1) as expected because habitat quality varied across landscapes, and variation between 500 × 500-m squares accounted for 23% – 30% of the variance in *pgr *in the general linear model (Table 1). It can be seen that carrying capacities broadly reflect the main features of the landscape shown in Figure. 1. Thus the primary habitats of the skylarks and spiders were the arable fields which occur in the triangular regions at lower right and top left, but there the voles and beetles were scarce. These distributions result from the known behavioural ecology of each species. Skylarks nest in arable fields because they prefer open and accessible low vegetation, and the modelled spiders are an opportunistic species specializing in disturbed habitats like arable fields. Voles prefer the permanent grass cover often found beside roadside verges and other linear features. The beetles prefer pasture and arable fields, but their numbers suffer in arable fields because of disturbance during farming operation [33].

Although carrying capacities varied, there were no differences between squares in the other key parameter, the return rate, except in the vole. This is shown by the parallelism of the *pgr*-density relationships, return rate being by definition the negative of the slope of this relationship. Invariance of return rates has also been reported in red kangaroos (*Macropus rufus*) in the pastoral zones in South Australia [18]. Invariance of return rates suggests, but does not prove, that the mechanisms of density dependence are the same in all squares.

Average return rates are shown in Table 2, and are less than one y^-1 ^for all four species, indicating that a return to carrying capacity after disturbance takes more than a year. These estimates are likely overestimates because our method of estimation of return rates has an intrinsic bias towards one [34], so the true values may be lower than shown in Table 2. Return rates less than one indicate that populations are stable and show no tendency to oscillate about their equilibrium values [3,35].

**Figure 4:**
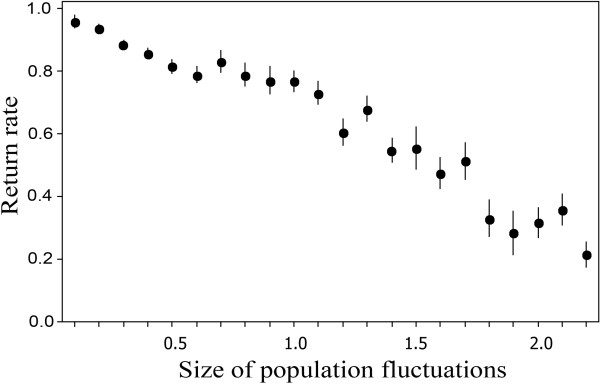
**Return rate in voles in relation to the size of within-square population fluctuations**. The size of population fluctuations within each square was assessed as maximum fold variation in population density [log_10_(*N*_max_/*N*_min_)]. Bars indicate standard errors.

The reasons why average return rates are less than one differ between species. The simplest cases are the beetles and the spiders, where we conjecture that the local populations are not able to recover from disturbance within a single season because the season is short and the larvae suffer high mortality even at low density, due to their locally patchy distributions. Both beetles and spiders have high fecundity but spiders recover from low density faster and have higher return rates than beetles (Table 2) because their juvenile stages escape density-dependent mortality by aerial dispersal (ballooning) [11,36]. By contrast beetle larvae do not disperse far [26] and so density remains patchy at small scales even in low-density years. The result is that beetle populations are slow to recover from low density and so have a lower return rate than spiders. Our interpretation here is supported by experimental manipulation of the dispersal characteristics of the beetle to resemble those of the spider, which resulted in the expected increase in return rate, to 0.85 +/- 0.01, y^-1^. The experiment only entailed increasing the maximum distance that beetle individuals could move in a day from 14 to 50 m, all other behavioural characteristics of the beetle were left unchanged. Return rates in voles are related to the size of within-square population fluctuations (Figure. 4). Return rates decline as the size of fluctuations increases (*r*_3214 _= -0.36, p < 0.001) from a value around one when fluctuations are small. The intermediary variable here is the size of the patch of habitat in which the voles live. In large patches adults compete for territories in contest competition and this results in a return rate of one, and little variation in numbers from year to year, because non-territorial animals remain within the patch moving in the interstices between territories. In small patches by contrast non-territorial animals are not able to hide and due to the large edge to area ratio must disperse outside the patch, where they usually die. This renders sub-populations in small patches vulnerable to extinction if the residents also die, after which population recovery is slow. Thus population fluctuations are higher and return rates are on average lower in smaller patches. Variation in return rates occurs in voles (*Microtus arvalis*) in Fennoscandia and eastern Europe [5,15], and it would be interesting to know if this is associated as here with the size of local population fluctuations.

The importance of our results lies in our quantification of the effects of spatial and temporal heterogeneity on the population dynamics of the four study species. The magnitude of these effects has implications for how we understand and predict population dynamics in reality. The effects of spatial and temporal heterogeneity must be accounted for if we are to have accurate predictive models for use in management and conservation. The size of the temporal effects shown here also has implications for how we evaluate long term temporal change (e.g. through climate change), since where there are large temporal variations, longterm changes will not be discernible quickly.

The effects of spatial heterogeneity were here shown by a significant square-density interaction, seen here in the vole (Table 1). Such interaction terms should be included in autoregression analyses of spatially separated populations, where return rate is given by the first autoregressive coefficient [5,6], but this has not been attempted to our knowledge except in [18]'s study of kangaroos. It is not possible to include all the interaction terms included here in analyses of real spatially separated populations, because there would not be enough degrees of freedom. In our simulations we overcame this by replicating the weather years. Nevertheless the interactions that were most important here could be included, these would be density*year and density*square. Together with the main effects of year and square this would allow analysis of whether both carrying capacities and return rates vary in space and time. This provides a method of answering the question as to whether there is spatial and temporal heterogeneity in the population's dynamics.

How general are our conclusions? There are several limitations to our study. The study species were selected because they represent different functional groups, and each has qualities that make them representative of other species, but more would certainly be better. Similarly other landscapes should be investigated. For example the landscapes experienced by northern Scandinavian voles are more homogeneous than those modelled here, so spatial heterogeneity should there affect population dynamics less. The effects of weather year are likely qualitatively robust, though quantitatively effects depend on the actual weather experienced. Lastly, we have not investigated the effects of population sizes in earlier years, *i.e*., *N*_*t*-1_, *N*_*t*-2_, but these would allow the identification of population cycles found in, *e.g*., some vole species [37,38].

It may be questioned as to whether we are here studying reality, or just very complex models. It is important to stress that our ABMs are the best available representations of life in the study site, which is why we chose to work with them. So, the emergent population properties of the ABMs should provide the most accurate characterisation of the real populations that is currently possible. Accuracy is not however guaranteed and checking – and correction – will probably be needed over the foreseeable future. The detailed nature of ABM predictions allows many checks, and such testing is ongoing. Related to this, it may seem surprising that not all emergent population properties of the ABMs are fully understood. Obtaining these mechanistic explanations is part of our research programme, however this can be laborious in ABMs as in reality, and success is not certain, as explained at the start of this Discussion.

## Conclusion

Here we have used previously published detailed ABMs to gain new conceptual insights into how populations behave in landscapes that vary geographically in realistic fashion. The results of much field work are encapsulated within each ABM but their population properties were not known in advance. The present study obtains population insights from known behavioural observations. The importance of our results lies in our demonstration that the effects of spatial and temporal heterogeneity must be accounted for if we are to have accurate predictive models for use in management and conservation. This is an area which until now has lacked an adequate theoretical framework and methodology [19,23,39].

## Authors' contributions

ALMaSS was created and modified as needed for the experiments reported here by CJT and the statistical analysis was performed by JN-N with RMS. All authors contributed to the conception and design of the study, to interpretation of the data, and to drafting and revising the article and all approve its publication.
